# Molecular Pathways of Carcinogenesis in Familial Adenomatous Polyposis

**DOI:** 10.3390/ijms24065687

**Published:** 2023-03-16

**Authors:** Ilaria Ditonno, Domenico Novielli, Francesca Celiberto, Salvatore Rizzi, Maria Rendina, Enzo Ierardi, Alfredo Di Leo, Giuseppe Losurdo

**Affiliations:** 1 Section of Gastroenterology, Department of Precision and Regenerative Medicine and Ionian Area, University of Bari, 70124 Bari, Italy; 2 Course in Organs and Tissues Transplantation and Cellular Therapies, Department of Precision Medicine Jonic Area, University “Aldo Moro” of Bari, 70124 Bari, Italy

**Keywords:** familial adenomatous polyposis, APC, cancerogenesis, microbiota, immune microenvironment, estrogen, chemoprevention

## Abstract

Familial adenomatous polyposis (FAP) is a genetic syndrome characterized by the presence of multiple polyps in the gastrointestinal tract and a wide range of systemic extra-intestinal manifestations. Patients affected will inevitably undergo abdominal surgery due to the malignant transformation of one or more adenomas. The pathogenesis of the disease is based on a loss of function mutation in adenomatous polyposis coli (APC), a tumor-suppressor gene, inherited following a Mendelian pattern. This gene is a key component of multiple cell functions that cooperate for homeostasis; when mutated, it contributes to the progression of colorectal adenoma into cancer. Recent studies have demonstrated that several additional mechanisms may influence this process, such as alterations in gut microbiota composition and mucosal barrier immunity, interaction with the immune microenvironment and inflammation, the hormone estrogen, and other signaling pathways. These factors represent promising targets of future therapies and chemoprevention, aiming to alter the progressive nature of the disease and improve the quality of life of families affected. Therefore, we performed a narrative review about the current knowledge of the aforementioned pathways involved in colorectal cancer pathogenesis in FAP, exploring the genetic and environmental factors that may contribute to the development of CRC in FAP.

## 1. Introduction

Familial adenomatous polyposis (FAP) is a hereditary cancer syndrome characterized by the presence of hundreds to thousands of synchronous polyps in the gastrointestinal tract, especially in the large intestine [1]. When untreated or undiagnosed, colorectal polyps will ultimately progress into colorectal cancer (CRC) when the patient reaches approximately 40 years of age [2]. Colorectal cancer remains a significant global health burden, representing the third most common diagnosed cancer in both sexes, although the sporadic form is steadily declining, presumably due to increased screening programs and the reduction of lifestyle risk factors in the general population [3,4]. Indeed, alcohol consumption, poor physical activity, obesity, high percentage of visceral fat and diet rich in red meat and poor in fibers are the main risk factors that are the most impactful on CRC prevalence worldwide, even in low-income and middle-income areas due to the process of Westernization [5]. Around 20% of patients have a positive family history, with at least one close relative (first- or second-degree relative) with a previous CRC diagnosis, showing nonsyndromic familial aggregation [6]. Instead, hereditary syndromes with a Mendelian inherited causative gene represent 5% of all CRC cases, among which FAP accounts for <1% [7]. Therefore, we performed a narrative review, whose scope was to explore the genetic and environmental factors that may contribute to the development of CRC in FAP.

## 2. Genetic and Molecular Aspects

The gene responsible for FAP is adenomatous polyposis coli (APC), a tumor-suppressor gene [8]. It is inherited following an autosomal dominant pattern, with an almost complete penetrance, equally affecting both genders. The prevalence is estimated to be 2.29 to 3.2 cases per 100,000 individuals [9]. Surprisingly, up to one third of cases are diagnosed in unidentified families, thus the proband has no affected relatives, representing cases of de novo mutations or mosaicism [10,11,12].

Except for the colorectal polyps, a hallmark of FAP, the disease spectrum is wide, with both additional gastrointestinal lesions, as fundic glands polyps in the stomach or periampullary adenomas in the small bowel, and extra-intestinal involvement, with the characteristic congenital hypertrophy of retinal pigment epithelium, which may be present at birth or in thyroid cancer, osteomas or dental abnormalities. As a consequence of increased survival rates, surveillance and management of extracolonic malignancies should be included in the patient follow-up [9,13].

The APC gene was first discovered in the 1970s, and, in the following years, follow-up studies deeply analyzed its structure and function [8]. It is located on chromosome 5, in the q21 region; it is a ubiquitous tumor-suppressor gene involved in the regulation of β-catenin in all tissues as part of the WNT signaling pathway, as it prevents constant activation of β-catenin that would bring uncontrolled cellular proliferation [14].

Moreover, APC also acts as a checkpoint protein in regulating the step from the G0/G1 to the S phase of the cell cycle. It encodes for more than 2000 amino acids, which accounts for 21 exons; the main part of the coding sequences is located on exon 15, which occupies a large portion of the gene [1]. Different regions of APC allow for different functions, with the proximal part responsible for oligomerization, the central part for regulation of WNT signaling pathway and cell cycle, and the C-terminal part for chromosomal segregation, cytoskeletal regulation and signal transduction [15]. Thus, according to the site in which the APC mutation occurs, a different portion of the protein will remain functional. Moreover, also the type of mutation will influence the residual activity of the protein; as a result, various clinical phenotypes will be encountered in clinical practice, varying from a mild gastrointestinal disease to multiple extra-intestinal complications.

The majority of inherited FAP mutations are clustered in the 5′-half of the gene. Mutations in an area called “mutation cluster region”, located on exon 15, between codon 1285 and codon 1580, are associated with the most severe disease phenotype, with the presence, among others, of desmoid tumors, hepatoblastoma and papillary thyroid carcinoma [16]. Mankaney et al. described in a retrospective study APC pathogenic variants in patients with and without upper gastrointestinal involvement affected by FAP; his group found an APC pathogenic variant to 5′ codon 1309 in 89% of patients with gastric cancer and sessile gastric polyps, with a cluster between codons 1061 and 1150 [17]. A milder form of the disease is present when APC mutations occur in the 3′ (codons 1581–2843) or 5′ region (codons 78–167) of the gene, called attenuated FAP (AFAP) [18]. Generally, these patients present with fewer than 100 colorectal polyps at an age older than patients with the classical variant, with an average age of CRC diagnosis at more than 40 years of age, usually in the absence of extra-intestinal features [7].

Several studies have demonstrated that the most common APC germline mutations found in patients with classical FAP are nonsense or frameshift mutations, detected in up to 80% of cases, which will produce an altered truncated protein [19]. There could be other different mechanisms responsible for disease-causing APC mutations, such as large deletions and duplications, splicing and missense alterations. New diagnostic methods, such as multiplex ligation-dependent probe amplification or next-generation sequencing, have now allowed the identification of such variants; their frequency was higher than expected in clinical practice, especially in patients with a previous negative genetic test [20]. Despite technological advances, we cannot completely diagnose all FAP cases, due to the difficulties in identifying further genetic alterations as mosaicisms or variations in deep intronic promoter regions [21].

**Figure 1:**
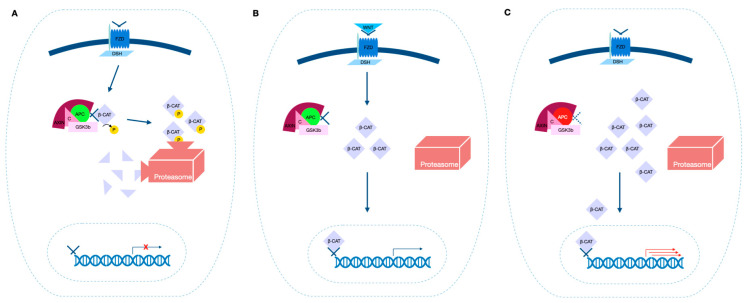
The simplified WNT/β-catenin pathway. (**A**) The WNT/β-catenin pathway in the absence of APC mutation (green) and without the ligand WNT: the “destruction complex” bounds, phosphorylates and targets β-catenin for ubiquitination; β-catenin is then degraded by proteasomes. (**B**) The WNT/β-catenin pathway in the absence of APC mutation (green) and with the presence of the ligand WNT: after WNT binds its receptor frizzled activating further intracellular molecules, β-catenin is no longer captured by the “destruction complex”; it accumulates in the cytoplasm and it migrates to the nucleus, where it acts as a transcription factor. (**C**) When APC is mutated (red), a faulty “destruction complex” is unable to bind and ubiquitinate β-catenin; as a result, it acts as an uncontrolled mitogenic signal, even in the absence of activation of the WNT signaling pathway by the ligand WNT. APC: adenomatous polyposis coli; C: conductin; GSK3b: glycogen synthase kinase 3 b; β-CAT: β-catenin; FZD: Frizzled receptor; DSH: Disheveled; P: phosphorylation.

In patients with FAP, a loss of function APC mutation will directly influence the WNT signaling pathway; when the proliferation signal mediated by WNT is absent, β-catenin is phosphorylated, bound to the “destruction complex”, namely a protein assembly made up of APC, axin (a cytosolic scaffold protein), conductin (a protein that phosphorylates serine and threonine residues of centrosomes) [22] and glycogen synthase kinase 3 b (GSK3b), and eventually degraded by proteasomes. On the other hand, when WNT binds the frizzled receptor, it activates a different protein cascade, which ends with GSK3b being inactivated [23]. Therefore, β-catenin is no longer destroyed and cells receive a proliferation signal; once stabilized, β-catenin migrates to the nucleus, where it acts as a transcriptional activator: it provides an uncontrolled permanent mitogenic signal, through the interaction with DNA-binding proteins (Figure 1) [24].

Beta-catenin also acts as a membrane adhesion molecule, forming a complex with E-cadherin, α-catenin and actin to stabilize the cell [25]. These interactions are disrupted when β-catenin is no longer degraded by the “destruction complex” due to APC mutations, especially when the region involved is the C-terminus [23]. When both APC (codons 1211–2075) and axin are phosphorylated by GSK3b, the binding of β-catenin to APC-axin complex is strengthened; as a consequence, the process of β-catenin degradation is enhanced [26].

The different functions altered in APC mutations may drive and promote CRC genesis. However, further additional mutations in other oncogenes or tumor-suppressor genes are necessary for the progression from early adenoma to invasive carcinoma. The process of tumorigenesis also in the case of FAP follows Knudson’s two-hit hypothesis, with the inactivation of both APC alleles; in FAP the first hit is represented by the inherited germline mutation [27]. Albuquerque et al. demonstrated that the two APC hits are not entirely independent from each other. The second hit is not selected randomly, in a way that the protein APC will retain a residual level of inhibition on β-catenin activity. Indeed, a completely uncontrolled β-catenin nuclear translocation, and the consequent uncontrolled replication, would induce cellular death and prevent tumor growth [28]. Therefore, the second hit in the APC gene follows the “just-right” signaling model, according to which the mutation allows a residual level of cell-cycle control. Beta-catenin is down-regulated by three 15-amino-acid repeats and seven 20-amino-acid repeats located on the APC gene; in the “just-right” model, some of them will remain functional in one of the two APC alleles, to partially control replication and avoid cell apoptosis [29,30].

Selective advantage was related to alterations in replication, segregation and survival, secondary to APC mutations acquired by the cells, initiates and drives tumorigenesis; further concomitant somatic mutations in other genes (i.e., KRAS, TP53) will follow as a consequence in a stepwise manner, accelerating the tumorigenic process along the adenoma to carcinoma sequence [23], just as for other non-hereditary tumors. Somatic APC mutations are also found in sporadic CRC cases. In this setting, an alternative pathway to gene inactivation occurs by the alteration of the APC promoter methylation, where hypermethylation down-regulates gene expression. However, such modifications have only been demonstrated in wild-type APC, while they are absent in already mutant (germline) APC [31]. Nonetheless, Devall et al. performed a DNA methylome-wide analysis of colon organoids derived from normal-appearing mucosal tissue of FAP patients and healthy subjects; they identified extensive differentially methylated regions between the two groups, some of which are also present in CRC [32].

The germline APC mutation, together with the progressive accumulation of mutations involving other oncogenes and tumor-suppressor genes, will cooperate to alter genomic stability. As mentioned, APC C-terminus domains are involved in chromosomal segregation during mitosis. More specifically, APC mutant cells have supernumerary centrosomes due to their altered duplication, and fail to connect spindle microtubules of dividing chromosomes to kinetochores, where APC localizes during the metaphase forming a complex with BUB1 and BUB3, two checkpoint proteins. These will affect chromosomes both quantitatively, with near-tetraploidy arising from non-disjunction defects, and qualitatively, with aberrant structural rearrangements, translocations from chromosome breakage and fragmentation due to multidirectional forces of spindles [15,23,33,34]. Such alterations give rise to chromosomal instability (CIN), a distinctive characteristic of cancers arising from APC mutations, as opposed to the other form of hereditary CRC with genetic instability such as microsatellite instability in Lynch syndrome. This additional role of APC on genome stability has been investigated by Samowitz et al., highlighting how APC and β-catenin mutations are not functionally equivalent despite both being associated with the constitutional activation of the WNT signaling pathway and initiation of tumorigenesis; the latter is not seen in large adenomas or invasive cancers, but is limited to small polyps. The additional tumor-suppressor functions of the APC gene, other than its interaction with β-catenin, are needed to promote carcinogenesis progression, together with synergistic interaction with the other oncogenes [35].

Multiple and diffuse adenomas in different stages of development are the hallmark of FAP; novel studies analyzing the genomic and transcriptomic profile of these patients have shed some light on the cancerogenic process. Li et al. compared whole-exome, whole-genome and single-cell RNA sequencing results of multiple adenomas at different stages of differentiation, adjacent normal tissues and carcinomas from FAP patients. They found that, in the same patient, adjacent spatially separated adenomas arose from the same cell, supporting the theory of field cancerization by crypt fission, although their results were limited by the restricted number of patients in the study [36]. This supports the hypothesis that the tumorigenic process starts before the formation of a visible lesion, which is even more dangerous in FAP patients as all their cells already harbor the inherited APC mutation favoring the process. By comparing the transcriptomic signatures in the normal colonic mucosa from FAP and sporadic CRC patients, they also highlighted an enhanced metabolic and proliferative activity in the specimens from FAP patients, probably resulting again from the altered APC [36]. Indeed, as metabolic reprogramming is a well-recognized hallmark of cancer, it is also increased in carcinomas of FAP patients and, to a lesser degree, in adenomas, guiding and sustaining the progression to invasive cancer.

## 3. Molecular Features of a Tumor Microenvironment

In recent years, novel insight in FAP pathogenesis and progression has been gained through human- and murine-based studies focused on the immune microenvironment (IME) role and composition, just as for many other diseases, especially for the potential of being a therapeutic target. The immune system is actively involved in controlling cancer progression and growth, as specific subsets of tumor-infiltrating lymphocytes, immune cells and cytokines are associated with increased survival. Different studies have focused on each component of the microenvironment, thus on the various immune cells, cytokines and chemokines, recently reviewed by Yang et al. [37]. Indeed, studies using Apc^Min/+^ mice performed by Tanner et al. have highlighted an altered CD4 and CD8 T-cell balance in number and function in lymphoid organs compared to wild-type Apc^+/+^ mice, modifying intestinal homeostasis and tumor immunosurveillance [38]. By contrast, in tissue samples from adenomas of FAP patients, CD4 T-cells have an increased number and an altered spatial distribution compared to controls, and CD8 T-cells are markedly decreased, as are B-cells in mice models. Both T regulatory cells (Tregs) and macrophages seem to mediate disease progression through an altered differentiation and function [37,39]. Natural killer (NK) cells so far do not seem altered qualitatively or quantitatively in the gut mucosa and the peripheral blood of FAP patients; however, further studies are needed. Indeed, Coletta et al. demonstrated lymphodepletion with subsequent reduction of B-cell progenitors and NK cells, due to an altered bone-marrow microenvironment in older Apc^Min/+^ mice carrying a specific APC germline mutation [37,40]. Moreover, other studies are also needed to investigate the role of the diverse NK effector cells in FAP progression, which is still largely unknown. Most of the secreted cytokines and chemokines have a dual function, either inducing or inhibiting cellular and tumoral growth, depending on the surrounding environment and other concomitant cells and molecules, representing a remarkable target for cancer immunotherapy worthy of further studies.

## 4. The Influence of Microbiota

The interaction between gut microbiota and the human host has recently been investigated due to its major role in development of colorectal polyps and progression to CRC, both in sporadic and hereditary settings, as well as its implication in the pathogenesis of other diseases not only restricted to the gastrointestinal tract [41]. The gut microbiota indicates a great number of microorganisms, comprising bacteria, fungi, archaea and protozoa, colonizing the human gastrointestinal tract since birth and coevolving symbiotically. The composition of microbiota is different among individuals and influenced by multiple factors such as diet, genetics, mode of delivery, use of different medications and others, and is continuously evolving over time [42,43]. It plays a part in many host physiological processes, regulating metabolism and nutrition, intestinal barrier function and response to pathogens mounting an immune response. An altered microbiota composition or imbalance, defined as dysbiosis, has been associated with different pathologies including cancer, even though whether it is a cause or a consequence is still unknown [43]. The adenoma to carcinoma sequence could be deeply influenced by some species of bacteria that contribute in many ways to polyposis progression. Indeed, different bacteria, altering their diversity indices and local distribution, interact with the human host and induce DNA damage and a prolonged inflammatory state that could initiate the carcinogenic process [41].

Liang et al. associated gut dysbiosis with APC mutations, analyzing the exon region of the APC gene and the metagenomics and metabolomics of stool samples of patients with intestinal adenomatous polyps [44], and linked specific species to an increased risk of polyp progression. In particular, they identified the association with *Faecalibacterium prausnitzii* and *Fusobacterium mortiferum*. Another study using Apc^Min/+^ mice highlighted how APC gene mutation could disrupt the interactions between the colonic mucosa and the microbiota, even prior to adenoma development, through the modification of the relative abundance of bacterial species [45]. Biondi et al. reviewed recent studies of CRC-associated bacterial strains, as *Fusobacterium nucleatum*, *Escherichia coli*, enterotoxigenic *Bacteroides fragilis* and *Campylobacter jejuni*, using Apc^Min/+^ mouse models, and their capacity to promote carcinogenesis through genotoxic damage, oxidative stress, inflammation, aneuploidy, abnormal cellular division and bacterial toxins [46]. Moreover, in patients affected by FAP, both adenoma and normal tissue specimens were marked by a panbacterial fluorescence in situ hybridization probe and showed a patchy bacterial mucus invasion and a bacterial biofilm mainly composed of *Escherichia coli* and *Bacteroides fragilis*, suggesting a link between bacteria harbored in colonic mucosa and tumorigenesis. It was hypothesized that APC mutations may influence bacterial interaction with the host by the enhancement of bacterial adhesion [47] (Table 1 [46]).

Furthermore, another feature that was encountered in FAP patients is an impaired cellular and mucosal barrier immunity, as proven by a reduction of resident memory T-cells and γδ T-cells in colonic mucosa and an increased IgA response to intraepithelial colonic microbes possibly related to an enhanced bacterial translocation. These changes allow a more prominent inflammatory process promoted by dysbiosis that may contribute to trigger and sustain tumorigenesis [55]. Microbiota modulations through probiotics, prebiotics or antibiotics, or even dietary interventions or fecal microbiota transplantation, may be a promising target of future therapies aimed at preventing or reducing polyp formation and their malignant degeneration, even if further studies and trials are needed.

## 5. Estrogen in FAP

Another molecule involved in the physiological and pathological mechanisms of the gastrointestinal system is the sex hormone estrogen; its effects are mediated by the binding of 17beta-estradiol (E2) to two types of nuclear estrogen receptors (ER), ERα and ERβ, which, after dimerization and translocation to the nucleus, induce the transcription of target genes. Both receptors are expressed in the normal colorectal mucosa, with a predominance of ERβ. The two isoforms induce opposite effects on cell replication and survival: the E2-ERα complex promotes cell survival and anti-apoptotic effects through the activation of multiple signaling pathways, and the E2-ERβ complex drives cells into apoptosis via p38/MAPK phosphorylation and the caspase-3 pathway [56,57]. Their expression has been evaluated in both sporadic adenomas and CRCs, with the identification of a marked reduction in the concentration of ERβ that could be already found in adenomas compared to controls, indicating its possible role in the early phases of carcinogenesis [58]. A similar analysis was performed on tissue samples from FAP patients who underwent colectomy: in hereditary cancers and polyposis, ERβ levels are reduced compared to healthy controls, with an even lower ERβ level compared to sporadic polyps [59,60]. Di Leo et al. obtained similar results analyzing duodenal polyps of FAP patients compared to controls [61]. The protective mechanism of estrogen in the adenoma to carcinoma sequence was investigated using Apc^Min/+^ mice models, which simulate the hereditary CRC pathway: male and female mice treated with an exogenous ERβ selective agonist exhibited a significant reduction in small-intestinal polyp number (39%) and diameter (36%) [62]. Another study demonstrated the protective role of endogenous estrogen, mediated by the up-regulation of ERβ and the down-regulation of ERα in the same mice models; indeed, tumors present in ovariectomized mice treated with E2 supplementation were equal in numbers to that of neither castrated nor supplemented with the hormone littermates [63]. Recent studies have demonstrated how tumors may be able to influence and communicate with the IME to their advantage through the action of extracellular vesicles, defined as lipid bilayer structures containing DNAs, proteins, mRNAs and microRNAs shed directly from tumoral cells; these structures are able to suppress an effective immune response and to induce tumor immunotolerance via transforming growth factor β (TGFβ) and Tregs [64]. Jiang et al. demonstrated that estrogen may contribute to counteracting the shaping of a favorable tumoral IME: in ovariectomized C57 mice injected with MC38 or CT26 mouse colon cancer cells, E2 supplementation reduced the levels of immunosuppressive molecules in tumor-derived extracellular vesicles, inhibiting their effect on Tregs in vitro, thus possibly reducing the immunosuppressive microenvironment and consequent antineoplastic effect [65].

The results obtained by both human and murine studies have pointed out a possible role for estrogen in cancer prevention, in both sporadic and high-risk individuals with hereditary CRC predisposition, and has also been proposed to be the basis of the gender-related difference in CRC risk seen in premenopausal women compared to age-matched men, which is abolished after the onset of menopause [66]. In this regard, phytoestrogens, plant-derived molecules with estrogenic-like features and able to bind ERs, have been evaluated in clinical and preclinical studies to assess their effects. Our group investigated how the addition of different types of phytoestrogens to the diet reduced the total number of adenomas and dysplasia as well as increased the rate of cellular death with a higher concentration of apoptotic markers (i.e., TUNEL, caspase-3) compared to controls on a high-fat low-fiber diet [67,68]. Calabrese et al. further proved these results in patients with a confirmed FAP diagnosis who underwent ileal pouch–anal anastomosis with a trial of a three-month active diet intervention based on a patented mixture of phytoestrogens and insoluble fibers [69]. Despite these promising in vivo and in vitro results, further and larger population-based studies and clinical trials are needed to confirm these results and to assess the onset of adverse effects derived from the activation of other estrogenic pathways in extra gastrointestinal tissues, especially in high-risk populations as FAP patients.

## 6. Counteracting Cancer in FAP

The progressive nature of this disease requires a constant need for endoscopic surveillance from late childhood or adolescence even after surgery, indicated when the disease is no longer manageable by endoscopic procedures due to the high burden of polyps, CRC or symptomatic polyps; moreover, patients also have to continuously monitor extra-intestinal disease manifestations [9]. Hence, the scientific community interest has been focused on the search for a chemopreventive agent to better manage the pathology from a systemic point of view and improve patient quality of life. One of the most studied pathways involved is cyclooxygenase (COX), more specifically in the isoform COX-2, a key enzyme involved in arachidonic acid transformation in prostaglandins and a target of common non-steroidal anti-inflammatory drugs. McLean et al. performed immunohistochemical assessment of COX-2 expression in sporadic colonic adenomas and paired normal mucosa; they identified a statistically significant increase of COX-2 in adenomas, with an even higher expression in polyps with a higher dimension and with a higher degree of dysplasia [70], thus with risk factors of malignant degeneration. The analysis of cells from APC-mutated zebrafish has linked APC to COX-2; in this model, the concentration of COX-2 was elevated, possibly due to the presence of an uncontrolled WNT/β-catenin signaling pathway [71]. Further studies confirmed the interplay between APC and COX-2 in animal models, using both genetically altered mice and pharmacological agents to inhibit COX-2 activity, showing a reduction in number and size of gastrointestinal polyps [72]. Zhang et al. analyzed the effects of COX-2 selective and non-selective inhibitors in vitro and in vivo on another molecular pathway that seems to be involved in FAP cancerogenesis with potential antineoplastic effects, namely the mammalian target of rapamycin (mTOR). Both drugs reduced mTOR signaling activity in CRC mice models, and their effect remained active even after COX-2 silencing with specific small interfering RNA, highlighting a COX-2 independent mechanism of suppression [73]. A study showed that COX inhibition may increase levels of NAG-1 which, in turn, enhances apoptosis [74], while sulindac, in a murine model of FAP, restored normal levels of apoptosis by down-regulating prostaglandin E2 [75]. Following research based on Apc^Min/+^, mice models have investigated the role of mTOR in FAP and confirmed its part in the cancerogenic process, as Bohan et al. reviewed [72]. It affects cellular growth by an increase in protein translation through an mTOR complex 1-mediated inhibition of eEF2 kinase [76]. Two human-based studies highlighted a successful use of sirolimus, an mTOR inhibitor, in reducing adenoma burden, as well as dysplastic degeneration, in FAP patients pre- and post-colectomy with ileal pouch anastomosis, making it possible to consider mTOR inhibitors as a promising potential chemopreventive agent [77,78].

## 7. Conclusions

**Figure 2:**
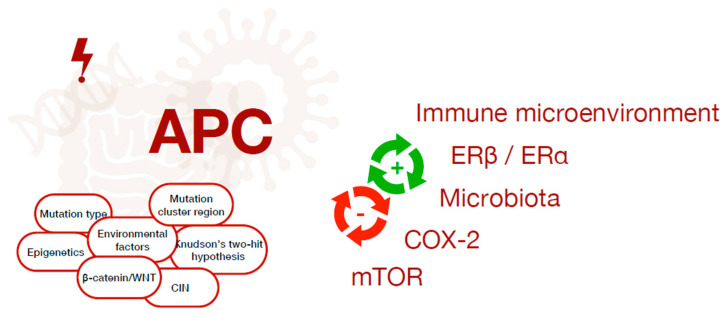
The complex interplay between several factors contributing to cancer onset in FAP patients, which depend on the interaction between genetic, environmental, hormonal, microbial and immunological factors.

Despite novel advances in the understanding of the molecular mechanisms here reviewed underlying the pathogenesis of FAP (Figure 2), the complete mechanisms are far from being defined. In the following years, a deeper understanding of the molecular biology and the genetics behind this disease may set the basis for novel therapies that could alter pathogenesis beyond intestinal polyps and their neoplastic degeneration, but also counteract the development of extra-intestinal neoplasms. Moreover, the role of microbiota and diet, inflammation and immune system needs to be further investigated and profoundly studied in randomized blinded trials as each of them may be a possible new target of future therapies.

## Figures and Tables

**Table 1:** Pathogenic bacteria in FAP mice models and patients.

Strain	Mechanism	Study Method	Ref.
*Faecalibacterium prausnitzii*	Altered abundance	FAP patients stool and serum samples were collected for metagenomics and metabolomics microbiota analysis	[44]
*Fusobacterium mortiferum*			
*Fusobacterium nucleatum*	Tumor-infiltrating myeloid cells	Infected Apc^Min/+^ mice	[48]
	TLR, NFκB, miRNA	CRC cell lines were incubated with *F. nucleatum* or control reagents, injected in mice models (including Apc^Min/+^) and analyzed in proliferation and would healing assays	[49]
	IL-6/p-STAT3/c-MYC via TLR4	Infection effect on macrophage polarization in human CRCs and cultured macrophages and the effects on macrophage phenotype and intestinal tumor formation in Apc^Min/+^ mice	[50]
	TLR4/PAK1 Cascade	Infected C57BL/6-Apc^Min/+^ mice	[51]
*Escherichia coli*	Colibactin	Germ-free Apc^Min/+^ and Apc^Min/+^;Il10^−/−^ mice were exposed to specific-pathogen-free or CRC-associated bacteria	[52]
	Biofilm	Polyps and macroscopically normal tissue of FAP patient were labeled with a panbacterial 16S rRNA fluorescence in situ hybridization probe, then specific pathogen-free wild-type AOM-treated mice were mono- or co-inoculated with strains identified in FAP patients	[47]
Enterotoxigenic *Bacteroides fragilis*			
	IL-17-dependent NFκB, STAT3, CXCL1	Infected Apc^Min/+^ mice	[53]
*Campylobacter jejuni*	Cytolethal distending toxin	Germ-free Apc^Min/+^ dextran sulfate sodium-treated mice colonized with human clinical isolate *C. jejuni*	[54]

## Data Availability

Not applicable.
